# Manganese distribution in the Mn-hyperaccumulator *Grevillea meisneri* from New Caledonia

**DOI:** 10.1038/s41598-021-03151-9

**Published:** 2021-12-10

**Authors:** Camille Bihanic, Eddy Petit, Roseline Perrot, Lucie Cases, Armelle Garcia, Franck Pelissier, Cyril Poullain, Camille Rivard, Martine Hossaert-McKey, Doyle McKey, Claude Grison

**Affiliations:** 1 Laboratoire de Chimie Bio-Inspirée et d’Innovations Écologiques, ChimEco, UMR 5021, CNRS-Université de Montpellier, Cap Delta, 1682 Rue de la Valsière, 34790 Grabels, France; 2 Institut Européen des Membranes, IEM-UMR 5635, ENSCM, CNRS-Université de Montpellier, 34090 Montpellier, France; 3 Service des Laboratoires Officiels Vétérinaires Agroalimentaires et Phytosanitaires de Nouvelle-Calédonie, DAVAR, New Caledonia, France; 4 Synchrotron SOLEIL, 91190 Saint-Aubin, France; 5 TRANSFORM, UAR 1008, INRAE, 44316 Nantes, France; 6 CEFE, Université de Montpellier, CNRS, EPHE, IRD, 1919 Route de Mende, 34293 Montpellier cedex 5, France

**Keywords:** Restoration ecology, Pollution remediation, Abiotic

## Abstract

New Caledonian endemic Mn-hyperaccumulator *Grevillea meisneri* is useful species for the preparation of ecocatalysts, which contain Mn–Ca oxides that are very difficult to synthesize under laboratory conditions. Mechanisms leading to their formation in the ecocatalysts are unknown. Comparing tissue-level microdistribution of these two elements could provide clues. We studied tissue-level distribution of Mn, Ca, and other elements in different tissues of *G. meisneri* using micro-X-Ray Fluorescence-spectroscopy (μXRF), and the speciation of Mn by micro-X-ray Absorption Near Edge Structure (µXANES), comparing nursery-grown plants transplanted into the site, and similar-sized plants growing naturally on the site. Mirroring patterns in other Grevillea species, Mn concentrations were highest in leaf epidermal tissues, in cortex and vascular tissues of stems and primary roots, and in phloem and pericycle–endodermis of parent cluster roots. Strong positive Mn/Ca correlations were observed in every tissue of *G. meisneri* where Mn was the most concentrated. Mn foliar speciation confirmed what was already reported for G. exul, with strong evidence for carboxylate counter-ions. The co-localization of Ca and Mn in the same tissues of *G. meisneri* might in some way facilitate the formation of mixed Ca–Mn oxides upon preparation of Eco-CaMnOx ecocatalysts from this plant. *Grevillea meisneri* has been successfully used in rehabilitation of degraded mining sites in New Caledonia, and in supplying biomass for production of ecocatalysts. We showed that transplanted nursery-grown seedlings accumulate as much Mn as do spontaneous plants, and sequester Mn in the same tissues, demonstrating the feasibility of large-scale transplantation programs for generating Mn-rich biomass.

## Introduction

More than 700 plant species worldwide are so far known to be hyperaccumulators of metals. These plants can accumulate heavy metals at levels 100 to 1000 times greater than in non-accumulating species, and are distinguished from “accumulators”, species that accumulate metals at high levels of without being a hyperaccumulator^1^. Ten metal and two metalloid (rare earth elements) elements are known to be hyperaccumulated, with reports for Ni being the most frequent (130 genera, 532 species), followed by Cu (43 genera, 53 species), Co (34 genera, 42 species), Mn (24 genera, 42 species) and Zn (12 genera, 20 species)^2^. Along with Cuba^3,4^, New Caledonia is considered to be a global metallophyte ‘hot-spot’^5^. As elsewhere, Ni-hyperaccumulators predominate, with 12 genera and 65 species^6^, but Mn-hyperaccumulators are also frequent (8 genera, 11 species)^7^. However, new hyperaccumulating species still remain to be discovered, as shown by recent research in this field^8,9^.

Metal hyper-accumulators are potentially useful resources in several contexts. They provide novel eco-catalysts with numerous industrial applications in ‘green’ and sustainable chemistry^10–14^. These polymetallic catalytic materials are used in organic synthesis to generate bio-sourced molecules with high added value and platform molecules as petrochemistry substitutes. Adapted to metalliferous soils, metal hyper-accumulators are also potentially useful in the revegetation of mining sites. However, despite the high number of reported (hyper)accumulating species, only a few have proven to be useful in the phytoremediation of mining sites. For example, although Ni-hyperaccumulators account for about 70% of known hyperaccumulators worldwide^2^, few of them are usable in mining restoration at large scale. *Alyssum murale* (Brassicaceae) has been successfully used in revegetation of Ni-contaminated mining sites in Oregon, USA^15–17^, but this European plant has also become an invasive species there^18^, demonstrating one advantage of using native plants in revegetation. In New Caledonia, our group demonstrated the potential of the endemic Ni-hyperaccumulator *Gessois pruinosa* (Cunoniaceae) in restoration after mining^19^. Accumulators of Cu and Co have been used in restoration of mining sites in Katanga Province, Democratic Republic of Congo^20^. Zn is another metal hyperaccumulated by some plants^2^. As shown by our work, they provide unique eco-catalysts^21,22^, but their generally low biomass and modest root systems limit their usefulness in revegetation^21^. Finally, 22 plant species are currently known to accumulate or hyperaccumulate rare earth elements (REE)^23^. REE-hyperaccumulators were discovered only recently, and so far have not been used in post-mining restoration.

Mn-(hyper)accumulating species have proved particularly suitable for use in revegetation of mining sites and valorization in “green” chemistry, for two reasons. First, they include trees, whose biomass production greatly exceeds that of Zn-, Ni- or REE-(hyper)accumulators. Second, of these metallic elements, Mn(II) is the only one to be in agreement with REACH regulation (Registration, Evaluation, Authorization and restriction of CHemicals), which aims at improving protection of human health and the environment from the risks caused by chemicals while promoting alternative methods^24^. Several Mn-(hyper)accumulating species have demonstrated their value in post-mining phytoremediation in New Caledonia^25–27^. These include three endemic species of *Grevillea* (Proteaeae); G. *exul* (subspecies *exul* and *rubiginosa*), *G. gillivrayi*, and *G. meisneri*. Like the endemic Ni-hyperaccumulating species, these Mn-(hyper)accumulating trees are highly adapted to the local climatic and edaphic constraints of mining sites^28–34^. These pioneer trees have been introduced with success on degraded sites characterized by open environments, frequent water stress, and poor soil^10^. With their substantial biomass and extensive root systems, these trees can effectively revegetate mine sites. They also accumulate large quantities of Mn, making them suited for applications in green chemistry. In ‘normal’ plants, foliar Mn concentration is around 50–800 μg g^−1^ dry wet (based on crop studies). In other species classed as Mn-accumulators, foliar Mn concentrations range from 3000 to 10,000 μg g^−1^^28,30,35^. In contrast, foliar Mn concentrations in Mn-hyperaccumulating species such as *Grevillea meisneri* can exceed 10,000 μg g^−1^^7,30,36^. Their Mn-rich biomass is easily valorized by the use of powdered leaves as green and highly efficient catalysts in organic chemistry^10,13,14,37^.

New Caledonia hosts 11 Mn-hyperaccumulators and 24 Mn-accumulators recorded so far^7,38^. However, a recent screening of herbarium samples in New Caledonia using a portable XRF instrument has revealed the existence of numerous new hyperaccumulators. Their number on the island stands now at about 196, and additional hyperaccumulating species doubtless remain to be discovered^8,9^. Apart from New Caledonia, other Mn-hyperaccumulating plant species are known from Australia^39,40^, Japan^41^, China^42,43^ and Malaysia^44^.

Mn is required by most organisms. In plants, its main role is as part of the Oxygen-Evolving Complex (OEC), co-factor of the Photosystem II (PSII) which performs the photo-oxidation of water during photosynthesis^45–47^. Mn plays also a main role in the activation of various enzymes in plants and as cofactors of enzymes, such as Mn-superoxide dismutase (Mn-SOD), Mn-catalase, pyruvate carboxylase and phospho-enolpyruvate carboxykinase^48^. In addition, Mn is involved in the biosynthesis of chlorophyll. Through its role in the activation of several enzymes in the tricarboxylic acid cycle in the shikimic acid pathway, Mn also plays a role in the biosynthesis of aromatic amino acids and of various secondary products, such as lignin and flavonoids^49^.

Some soils are Mn-deficient^50^ and Mn-deficiency strongly affects photosynthesis^51^. In contrast, in some soils Mn reaches levels that are toxic for many plants^52^. Excess of Mn is particularly damaging for the photosynthetic apparatus^53^ but can also induce oxidative stress by the formation of reactive oxygen species^54^.

Adapting to such variation may require fine regulation of Mn assimilation and transport mechanisms^55,56^. Some plants are able to grow in soils in which other plants suffer Mn toxicity. Two mechanisms can account for such resistance: avoidance and tolerance^57^. One proposed mechanism of tolerance, which means the ability to tolerate high Mn concentrations in tissues, involves an efficient transport and sequestration of Mn in tissues where it does not interfere with metabolic activities. Thus, much attention has been devoted to studying the tissue microdistribution of metal elements in plants that hyperaccumulate them^55^.

Most previous studies on metal hyperaccumulators have focused on the distribution of metals within leaves, using various in vivo microprobe localization techniques. Pioneer studies conducted on Ni, Zn and Cd concluded that the highest foliar concentrations of these metals occurred in non-photosynthetic tissues^58–64^.

In contrast, the first investigation of a Mn-hyperaccumulating species by microprobe techniques, conducted on *Gossia bidwillii* (Benth.) N. Snow & Guymer (Myrtaceae), revealed an unusual sequestration pattern, with Mn being mostly concentrated in the palisade mesophyll cells^39^. Subsequent studies of species in several families demonstrated three different patterns of Mn sequestration, with higher concentrations in dermal layers^65–69^ or in the palisade mesophyll^70–74^, or with Mn homogeneously distributed throughout the leaf cells^68^. Up to now, such variability in the foliar distribution of metals has been reported only in Mn-hyperaccumulators.

The Eco-Mn catalysts derived from the leaves of endemic New Caledonian Mn-(hyper)accumulators—three of four *Grevillea* taxa, including *G. meisneri*, as well as *Garcinia amplexicaulis*—exhibit a remarkable feature: the presence of mixed Ca–Mn oxides, Ca_2_Mn_3_O_8_ and CaMnO_3_^27,75^. These Eco-CaMnO_x_ catalysts have distinct catalytic behavior making them suited for several novel applications^75^. Synthesis of these mixed oxides is difficult to achieve under laboratory (and industrial) conditions^76^, and its presence in the ecocatalysts derived from the leaves of these plants is a significant discovery. The formation of these oxides may be inherently dependent on the physiology of these plants, but mechanisms are unknown. Describing the patterns of distributions of different metal elements in tissues of living hyperaccumulator plants may provide clues to understanding the interactions between them in the preparation of ecocatalysts.

Only one species of *Grevillea*, *G. exul*, has previously been studied by in vivo microprobe methodologies^68,77–79^. The first aim of the present study was to characterize the fine-scale tissue distribution of Mn in *Grevillea meisneri*, the only New Caledonian Mn-hyperaccumulator of the genus. The second objective was to determine whether the distributions of different metal elements are correlated (positively or negatively) with one another. We were particularly interested in the co-distribution patterns of Mn and Ca, in the hope of obtaining clues about the mechanisms leading to the formation of mixed Mn–Ca oxides in the ecocatalysts prepared from Mn-(hyper)accumulators. Although such mixed oxides are present in ecocatalysts prepared from prepared from different Grevillea species and *Garcinia amplexicaulis*^27^, we focused on *Grevillea meisneri*, as its performance shows the greatest potential for use in revegetation and in supplying biomass for production of ecocatalysts. A subsidiary objective of the study was to determine whether transplantation of nursery-grown *G. meisneri* seedlings into contaminated mining sites is an effective seeding strategy for revegetation efforts.

## Material and methods

### Study site

The study was conducted in degraded mining sites in ‘Creek à Paul’ valley near Tiébaghi (20°29′35.03″S, 164°12′22.76″E), New Caledonia. Prior to mining operations, the valley’s soils were brown hypermagnesian soils, classified as Magnesic Cambisols^80^. Derived from ultramafic rocks, these soils are characterized by low concentrations of essential elements, particularly P, high concentrations of heavy metals such as Ni, Mn, Co, and Cr, and a high ratio of exchangeable Mg/Ca^81^. During mining operations, topsoil was removed, along with most of the organic matter and mineral nutrients, leaving behind a gravelly substrate composed of mining residues rich in transition metals, including Ni, Mn, and two highly toxic elements, Cr and Co. Concentrations of nutrient elements (Ca, K, and especially P) are even lower than in the undisturbed soil prior to mining, whereas Mg can reach toxic levels. Organic matter is present in very small quantities^26,82^, and soils developing in this substrate are sterile and N-deficient. Along with these highly constraining chemical properties, post-mining soils have been compacted by heavy machinery and present physical properties inimical to plant growth. Furthermore, in this seasonally dry climate, the open conditions are unsuitable for the establishment of forest species. All these conditions combine to make spontaneous revegetalisation very difficult, if not impossible^31,82^. Soils composition on the mining sites in ‘Creek à Paul’ are given in Table S1.

### Study species: *Grevillea meisneri*

Endemic to New Caledonia^83^, *G. meisneri* grows only on the northwestern coast of the ‘Grande Terre’, on ‘maquis’ of variable density, preferentially on brown hypermagnesian soils under 650 m altitude^84^. *Grevillea meisneri* is the only Mn-hyperaccumulator among the *Grevillea* species endemic to New Caledonia^7^. This species was chosen for restoration efforts because of its adaptations to the site’s harsh climatic and edaphic constraints. Observations of *G. meisneri* in its natural habitats indicated a good ability to establish.

**Figure 1:**
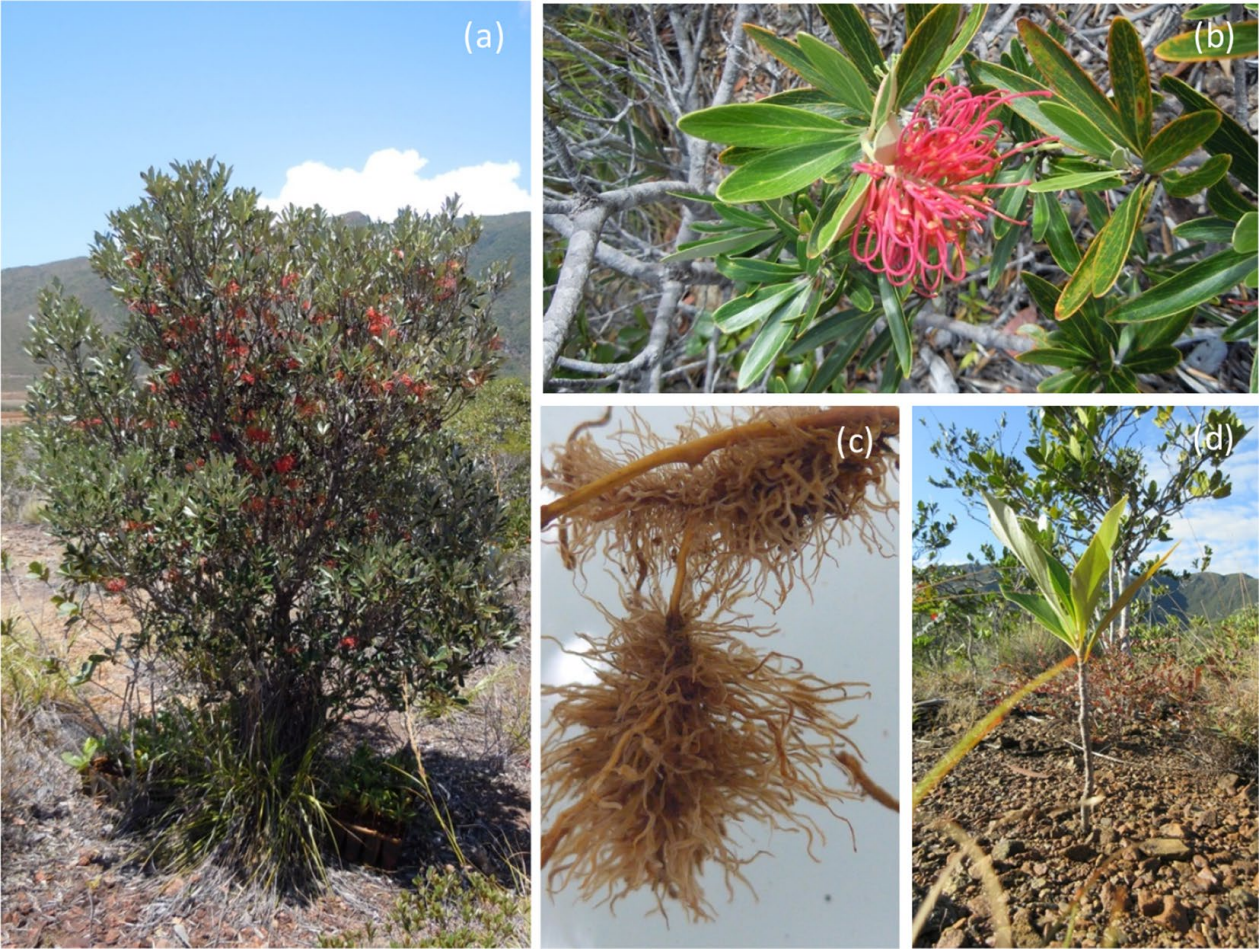
(**a**) Mature specimen of *Grevillea meisneri*; (**b**) Close-up of the inflorescence and the leaves of *G. meisneri*; (**c**) Close-up of the cluster roots of *G. meisneri*; (**d**) A young specimen of *G. meisneri* used for this study.

*Grevillea meisneri* is a shrubby species that can reach up to 7 m height (Fig. 1a). Its tortuous branches are glabrous, rubiginous when young, and the wood is ash grey. The leaves are simple, coriaceous, lanceolate to elliptic, 35–140 mm long × 6–70 mm wide, petiolate. The adaxial surface (hereinafter referred to as ‘upper epidermis’) is glabrous and shiny light-green, whereas the abaxial surface (hereinafter referred to as ‘lower epidermis’) is hairy, matte and brownish, even silver-grey. The entire margin is entirely recurved underneath and the fine pinnate venation is barely visible. The brightly coloured carmine-red flowers are clustered into simple, cylindrical and pendulous racemes at the end of branches (Fig. 1b)^85^. Like other Proteaceae, *G. meisneri* is non-mycorrhizal but, like almost all other members of the family, develops cluster roots that improve nutrient uptake (Fig. 1c)^86–89^.

### Plant materials

Restoration was carried out using plants of *G. meisneri*, among other (hyper)accumulators of metals (e.g. Ni, Zn). In 2014, 450 plants of *G. meisneri* were obtained from seeds collected in populations in the North Province of New Caledonia and grown for 18 months in pots in a nursery (see Supporting methods). Soil used in these pots was topsoil of brown hypermagnesian soils, collected in the valley of “Creek à Paul” prior to mining operations. The 18-month-old seedlings were then introduced into the post-mining site. Soil of each planting hole was amended with Yates nutricote 365, Superphosphate triple and a water-retention agent. The composition and quantity of fertilizers used for the plantation are given in Tables S2–S4. No further treatment was made post-plantation. Use of transplanted plants in restoration efforts was considered a challenge, because the site’s edaphic and climatic conditions make establishment difficult, even for species such as *G. meisneri* that are adapted to local climate and to metal-rich soils. However, we considered that the performance of direct seeding would likely be even poorer.

For comparative purposes, we examined elemental composition in tissues of a *Grevillea* taxon that is neither a New Caledonian endemic nor a hyperaccumulator of Mn. A commercially available ornamental variety known as “*Grevillea* ‘rosa jenkinsii*’ ”*, derived from the Australian species *Grevillea rosmarinifolia* A. Cunn. Plants were grown in Montpellier under conventional conditions.

### Harvesting of plant samples

Whole living plants of *G. meisneri* were collected in 2019. Dr C. Poullain undertook the formal identification of the plant material used in our study. Plant material collection and authorization for publication are according to the regulation of the North Province no 609011-40/2019/DEPART/JJC with effect since July 8th, 2019. This authorization is specific to this study and concerns the harvest of whole plants in the valley of “Creek à Paul” for analysis purpose of vegetal tissues. It was based on the Governmental authorization 036/2019, effective from June 6th, 2019 to June 5th, 2020. It follows the convention no 53 from October 18th, 2016 that stipulates New Caledonia authorizes the CNRS to have access to the entire private domain available of New Caledonia for the harvest of biological resources for purpose of scientific studies on vegetal species capable to hyperaccumulate heavy metals. The regulation on the access and collecting of biological, genetic and biochemical resources follows the Convention on Biological Diversity (CBD) and the Conservation of Migratory Species (CMS).

Transplanted plants, and spontaneously growing specimens of the same height and living in the same valley, were selected (Fig. 1d). Plants were collected with their soil and kept in pots for transportation to the laboratory, where samples were prepared within 24 h maximum post-harvest.

Measurements, shapes and colours of the different organs are based on the examination of herbarium material, fields, observations and material collected. The herbarium specimens of Grevillea were examined with the herbarium of Nouméa (NOU) (Herbarium acronyms follow Thiers 2020). The collections of *G. meisneri* from the north east of the main island were characterized by their architecture, the inflorescence axis, pedicels and perianth (Girardi plate 59 of the NOU herbarium). *G. meisneri* is easily distinguished by its vertically pendant inflorescences with a curved axis (rather than more or less horizontal inflorescences with a straight axis) and by its leaves less than 12 cm long on sterile axes^83^. A voucher specimen of *G. meisneri* from the site has been deposited in the herbarium of Nouméa (NOU) with the deposition number POU-0373.

### MP-AES analyses

Tissue samples including leaves, stems, roots and cluster roots of *G. meisneri* were carefully washed with deionized water to remove soil particles and then oven-dried at 60 °C overnight before being ground. Mineral composition was determined using an Agilent 4200 Microwave Plasma-Atomic Emission Spectrometer (MP-AES) coupled with an SPS4 autosampler. Approximatively 20 mg of the ground biomass was digested in 6 mL of reversed aqua regia (1:2 hydrochloric acid (37%): nitric acid (65%)) under an Anton Paar Multiwave Go microwave-assisted digestion, with the following program: 20–164 °C in 20 min then 10 min isothermal at 164 °C. Samples were filtered and then diluted to 0.2 g L^−1^ in nitric acid (1%). Three blanks were recorded for each step of the dilution procedure. Three analyses of the mineral composition were carried out for each sample to check repeatability of the measurement.

### Ion chromatography

Ion chromatography analyses were carried out with a Metrohm 882 Compact IC instrument equipped with chemical (Metrohm suppressor MSM II for chemical) and sequential (Metrohm CO_2_ suppressor MCS) suppression modules, and a conductivity detector. Separations were performed on a Metrosep A Supp 16-250/4.0 column associated with a guard column kept at 55 °C. The mobile phase consisted of a mixture of 7.5 mM Na_2_CO_3_ and 0.75 mM NaOH in ultrapure water. The flow rate was 0.8 mL min^−1^. Standard solutions were prepared from a commercial multi-element standard solution of 1000 μg mL^−1^ of F^−^, Cl^−^, Br^−^, NO_3_^−^, PO_4_^−^ and SO_4_^2−^ and from Na_2_C_2_O_4_ and C_4_H_4_O_5_Na_2_. Analyses were carried out at Laboratoire de Chimie Physique et Microbiologie pour les Matériaux et l’Environnement (LCPME—Université de Lorraine, France). Two separate chromatography analyses were recorded for each sample.

The samples were prepared using fresh leaves that were ground in deionized water using a mortar and pestle. The homogenate was filtered and the filtrate was collected for analysis. The residue was then extracted with isopropanol in the same way. The resulting residue was then finally extracted with dichloromethane, following the same procedure. The three filtrates were collected and analyzed.

### Light microscopy

Fresh samples of *G. meisneri*, including leaves, stems, roots and cluster roots, were collected in situ and immediately fixed in formalin (10%) during 24 h. Fixed tissues were then embedded in paraffin (Paraplast plus) using a SAKURA VIP^®^ 5 JR vacuum infiltration processor and a SAKURA TEC-5 embedding console. The samples were sectioned at 6 µm thickness under a rotary microtome (Leica RM2245). After staining with carmine red/iodine green, the tissue samples were examined using a light microscope (Leica DM3000) equipped with a camera (Leica MC170 HD).

### Sample preparation for μXFM analyses

Leaves, stems, roots and cluster roots of living plants of *G. meisneri* were carefully washed with deionized water before being dried with absorbing paper. Samples were cut and fragments of about 1 cm long were immediately immersed in an embedding compound (Optimal Cutting Temperature OCT from VWR) and fast frozen in isopentane, as liquid cryogen, cooled by liquid nitrogen^90^. This cryofixation protocol ensured an extremely fast cooling of the samples, preventing ice crystal formation. Therefore, it limited damage to the cellular structures and preserved elemental distribution close to its natural state. Leaf samples were divided into mid-rib (central vein) and margin. For the roots, the primary root and cluster roots were sampled. The samples were transported from New Caledonia in a cryogenic container, kept at − 80 °C using dry ice, to SOLEIL Synchrotron (Saint-Aubin) for analysis and kept in a freezer at − 80 °C before cryo-sectioning.

Transverse sections of frozen-hydrated tissues were cut at a thickness of 20 μm using a cryo-microtome (Thermo Fisher Scientific). The cryo-chamber was kept at − 20 °C. The thin sections were placed between two Ultralene films (SPEX SamplePrep) in a copper sample holder.

### Synchrotron μXRF and μXANES

The micro-X-ray fluorescence microscopy (µXRF) and micro-X-ray Absorption Near Edge Structure (μXANES) analyses were carried out at the LUCIA beamline^91^ at SOLEIL Synchrotron. The X-ray beam was monochromatized at 6.65 keV using a fixed exit double-crystal Si (111) monochromator, calibrated to 6539 eV at the first inflection point of a thin elemental Mn foil XANES spectrum. The beam was focused to 3.6 × 2.9 (v × h) μm^2^ by means of a Kirkpatrick-Baez mirrors arrangement. The XRF signal was collected using a mono-element (60 mm^2^) Bruker silicon drift diode detector. Samples were transferred into the experimental chamber under liquid nitrogen vapor to maintain the cold chain. Samples were scanned under cryogenic conditions using a liquid nitrogen cryostat, and under vacuum in order to limit absorption and scattering by air. µXRF maps were collected in continuous Fly-scan mode with a pixel size of 3 × 3 μm^2^ and an integration time of 200 ms per pixel for high spatial resolution maps. The selection of appropriate areas for high spatial resolution maps was based on low spatial resolution maps (pixel size between 10 × 10 and 100 × 100 μm^2^ and with an integration time of 50 ms per pixel). µXANES spectra were collected on points of interest, based on the foliar µXRF elemental maps. Continuous FlyScan XANES were recorded in fluorescence mode in the 6475–6700 eV energy range with an equivalent energy step of 0.2 eV and an integration time of 100 ms per point. One to four spectra were collected on the same points of interests, as a function of the Mn concentrations.

### Data processing and statistics

The count number for the XRF signal was normalized by the count number of the incoming beam signal and corrected by the XRF detector dead time. To optimize the discrimination of the various XRF line contributions, the XRF signal of each element was extracted by batch fitting the XRF spectrum in each pixel of the map using the PyMCA software^92^. The XANES spectra were normalized and background-subtracted using Athena software^93^ and the repetitions merged.

### Data transformation and statistical analyses

Regions of interest (ROIs) were selected from the maps using the ROI tool in the Fiji software^94^ according to the tissues of the organs. The different types of tissues identified are reported in Table 1. Average fluorescence intensities of each element among the different tissues were obtained from the selected ROIs.

**Table 1:** Tissues identified in the different organs of *Grevillea* *meisneri*. Tissues were described from the outside to the inside of the corresponding organ.

Organ	Identified tissues
Leaf margin	Cuticle, upper epidermis, palisade mesophyll, spongy mesophyll, lower epidermis
Leaf mid-rib	Upper epidermis, palisade mesophyll, vascular bundles, spongy mesophyll, lower epidermis
Stem	Periderm, cortex, phloem, xylem, pith
Primary root	Periderm, cortex, phloem, xylem
Parent root and cluster rootlets	Cluster rootlets, epidermis, cortex, periderm and endoderm layers, phloem, xylem

Correlations between Mn, Ca, Cl, K, Mg, P and S concentrations were estimated by extracting fluorescence intensity pixel by pixel for each element from the selected ROIs. Pearson correlation coefficients between the different elements, and their P-values, were calculated using R (R Core Team). Holm’s correction for multiple comparisons tests was applied.

### PCA analyses

Principal component analyses (PCA) were performed for each element on judiciously chosen tissues of the different organs of *G. meisneri* to investigate co-localisation of the elements, with a particular focus on Mn. Data used for PCA analyses were collected from the tissues of each organ in which Mn was most highly concentrated, i.e., the dermal layers for the leaf, the cortex for the stem, the cortex of the primary root, and the pericycle–endodermis layers and phloem of the parent cluster root. Unscramble (Camo Analytics) was used to perform the PCA.

## Results

### Bulk elemental composition in tissues of *Grevillea meisneri*

The elemental composition of tissues of *G. meisneri* is shown in Table 2. Calcium was the major mineral element in all plant tissues analyzed, with a range of concentrations of 0.5–0.8 wt % (DW). Manganese presented concentrations similar to or even higher than those of macronutrient elements such as K and Mg in each tissue and especially in leaves, with up to 0.4 wt %. No significant differences in concentrations of Mn were observed between nursery-grown plants that had been transplanted and those that had grown spontaneously in the rehabilitation site. Although soils of the site are enriched in Ni, this element was not detected in any part of *G. meisneri* plants. As a comparison, the cultivated variety known as “*Grevillea* ‘rosa jenkinsii*’ ”*, derived from the Australian species *Grevillea rosmarinifolia* A. Cunn., presented quite similar foliar composition in macronutrient, such as K, Mg, Na… but higher Ca concentrations than in *G. meisneri*. The very low concentrations of Mn in their leaves, less than 0.1 wt%, testified *Grevillea* ‘rosa jenkinsii*’* is not an accumulator of Mn compared to New Caledonian Grevillea species.

**Table 2:** Elemental composition in tissues of plants of *Grevillea meisneri* that had been grown in a nursery and then transplanted (T) and plants that had grown spontaneously (S) in the rehabilitation site, determined by MP-AES analyses (mean ± standard error).

Tissues	Group	Composition [wt % (± S.E.)]				
		Mn	Ca	K	Mg	Na
Leaves	T (7)	0.31 (± 0.09)	0.59 (± 0.07)	0.30 (± 0.07)	0.23 (± 0.06)	0.12 (± 0.03)
	S (8)	0.39 (± 0.21)	0.55 (± 0.16)	0.29 (± 0.11)	0.11 (± 0.04)	0.18 (± 0.10)
Stems	T (4)	0.14 (± 0.05)	0.63(± 0.19)	0.35 (± 0.12)	0.13 (± 0.06)	0.09 (± 0.04)
	S (2)	0.22 (± 0.08)	0.50 (± 0.04)	0.10 (± 0.08)	0.17 (± 0.04)	0.15 (± 0.02)
Primary roots	T (6)	0.06 (± 0.03)	0.24 (± 0.08)	0.11 (± 0.09)	0.14 (± 0.04)	0.08 (± 0.02)
	S (6)	0.20 (± 0.10)	0.74 (± 0.25)	0.20 (± 0.11)	0.23 (± 0.20)	0.16 (± 0.06)
Parent cluster roots	T (6)	0.12 (± 0.03)	1.00 (± 0.28)	0.31 (± 0.18)	0.29 (± 0.06)	0.43 (± 0.09)
	S (2)	0.21 (± 0.11)	0.49 (± 0.02)	0.11 (± 0.08)	0.44 (± 0.36)	0.22 (± 0.01)

Numbers in brackets denote the separate samples analyzed. Ni was also analyzed but not detected.

Concentrations of P and S in leaves were determined by ICP-MS (Table S6). The concentration of P was very low, even lower than the concentration of S, as in other native New Caledonian *Grevillea* spp. Conversely, *Grevillea* ‘rosa jenkinsii*’* presented a much higher P concentration (Table S6).

### Ion chromatography of *Grevillea meisneri* leaves

Chemical composition of the leaves of *G. meisneri* was further analyzed by ion chromatography (Table S7). The presence of carboxylates was investigated and analyses revealed high contents of malate and oxalate, while citrate could not be detected. Two other anions were abundant, sulfate and phosphate. Concentration of phosphate was about four times higher than that of sulfate. This high phosphate content is surprising for a plant belonging to the family Proteaceae, growing in such phosphorus-depleted soil.

### Anatomical features of *Grevillea meisneri* leaves, stems and roots

Light microscopic analysis was conducted on leaves, stems and roots of *G. meisneri*. The different types of tissues identified in the various organs of *G. meisneri* are reported in Table 1. Using the criteria defined in our methods (see Supporting methods)^95^, no differences were observed in anatomical features of plants that had been transplanted and those that had grown spontaneously in the rehabilitation site.

**Figure 2:**
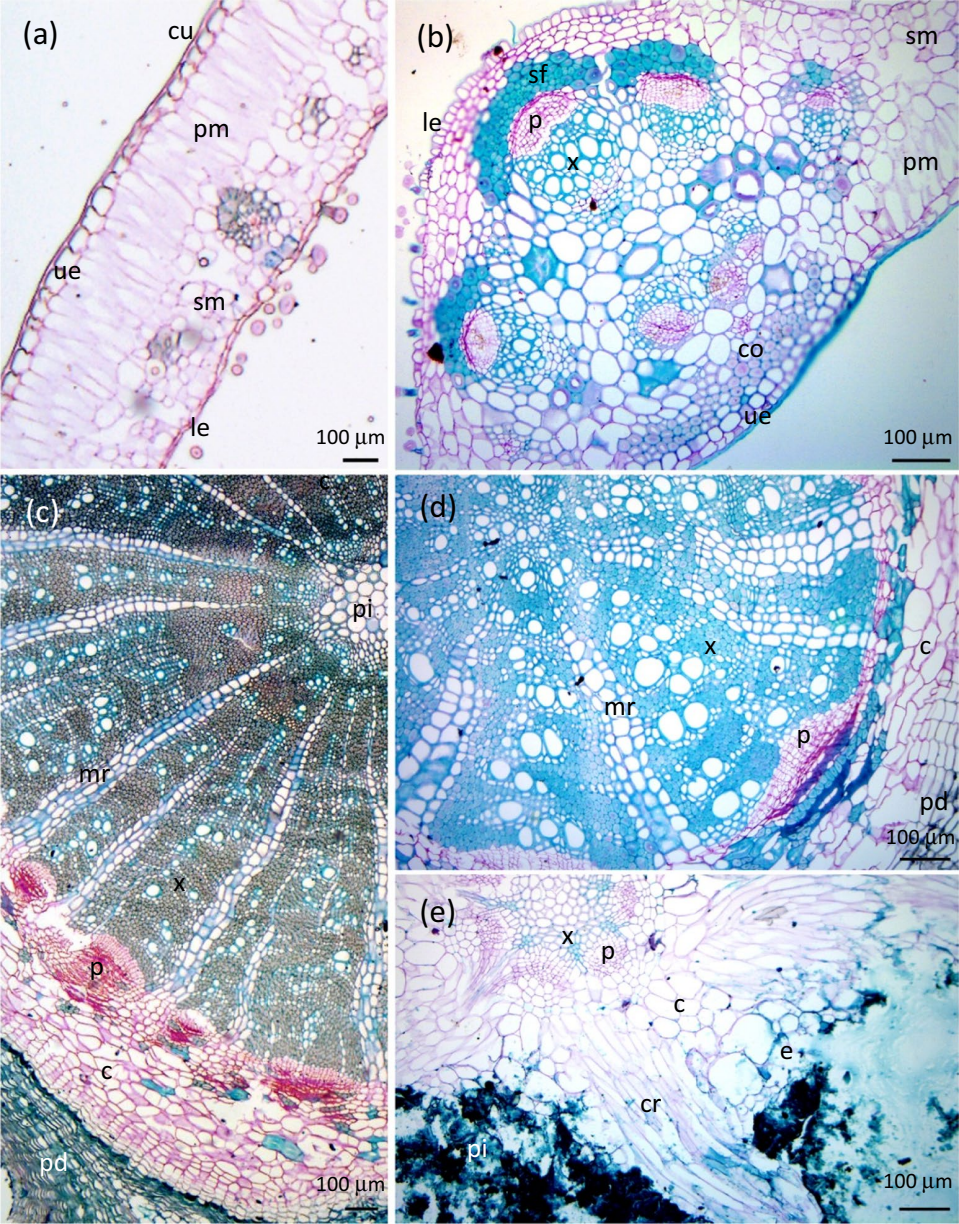
Light microscopy images of *Grevillea meisneri* anatomical structures. (**a**) Margin of a leaf; (**b**) mid-rib of a leaf; (**c**) stem; (**d**) primary root; (**e**) secondary root surrounded by the rootlets of a cluster. *c* cortex, *co* collenchyma, *cr* cluster rootlet, *cu* cuticle, *e* epidermis, *le* lower epidermis, *mr* medullary ray, *p* phloem, *pi* pith, *pm* palisade mesophyll, *pd* periderm, *sf* sclerenchyma fiber, *sm* spongy mesophyll, *ue* upper epidermis, *x* xylem.

Light microscopy images of leaves showed a thick cuticular layer, as expected for a xerophytic species (Fig. 2a,b). The upper epidermis is composed of a single layer of large cells, twice as large as those of the lower epidermis. The palisade mesophyll consists of thin, elongated cells aligned in a single layer, as was observed using PIXE/EDS and SEM/EDS for *Grevillea exul*^68^. The spongy mesophyll accounted for more than half of the total thickness of the mesophyll. The dermal layers accounted for about 25% of the total leaf volume.

The stems exhibited a typical secondary growth structure with a small cortex, a large stele with well-defined vascular bundles, and a periderm, which comprises the outermost tissue of the stem (Fig. 2c). Likewise, the primary roots had a typical secondary growth structure (Fig. 2d). Samples of cluster roots were cut so as to obtain a cross-section of the parent root, so only a tangential section of cluster rootlets stemming from the parent root could be mapped (Fig. 2e). Light microscopy images of cluster roots clearly revealed the primary structure of the parent root, with a wide parenchymatous cortex split by cluster rootlets. The rootlets seemed to emerge from the parental pericycle, opposite the xylem poles, as first described by Purnell (1960) for the general structure of cluster roots^86^. The tangential section of the cluster rootlets gave little information concerning the anatomical structure of the rootlets.

### Elemental distribution in leaves, stems and roots of *Grevillea meisneri* determined by μXRF

Micro-X-ray fluorescence was performed on leaves, stems, roots and cluster roots of transplanted and spontaneously growing plants of *G. meisneri* and allowed elemental mapping of frozen hydrated tissues in a state as close as possible to the native state^72^. The μXRF maps of *G. meisneri* leaves, stems and roots showed no differences in elemental distributions between transplanted plants and those growing spontaneously in the site. μXRF maps obtained for *G. meisneri* spontaneously growing in the site are given in Supporting Information. The brightness of each elemental μXRF map was scaled for an optimum contrast, so the pixel intensities did not represent relative differences in concentration between different elements, but represented differences in concentration for a given element in the different maps. μXRF maps of K were used as a control since the presence of cellular K in all samples indicated the retention of cell contents, thus showing the efficiency of the preparation.

**Figure 3:**
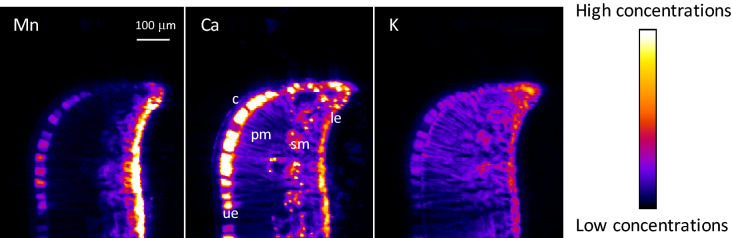
μXRF maps of Mn, Ca, and K for a cross-section of a frozen hydrated leaf margin of transplanted *Grevillea meisneri*. The pixel size is 3 μm. The intensity scales are different between elements. *c* cuticle, *le* lower epidermis, *pm* palisade mesophyll, *sm* spongy mesophyll, *ue* upper epidermis.

μXRF maps of the leaves revealed that Mn was sequestered in the epidermis, mostly in the lower epidermis, and was found, in lower concentrations, in vascular tissues and the spongy mesophyll (Fig. 3 and Figs. S1–S4). Ca was also mostly localized in the dermal layers, with a higher concentration in the upper epidermis. High-intensity Ca spots throughout the spongy mesophyll suggested the presence of calcium oxalate crystals. Magnesium was mostly localized in the dermal tissues. Other nutrient elements (K, Cl, S and P) were globally distributed throughout the leaves. However, P showed a higher concentration in the vascular tissues, whereas S was mostly in the mesophyll (Figs. S2 and S4).

**Figure 4:**
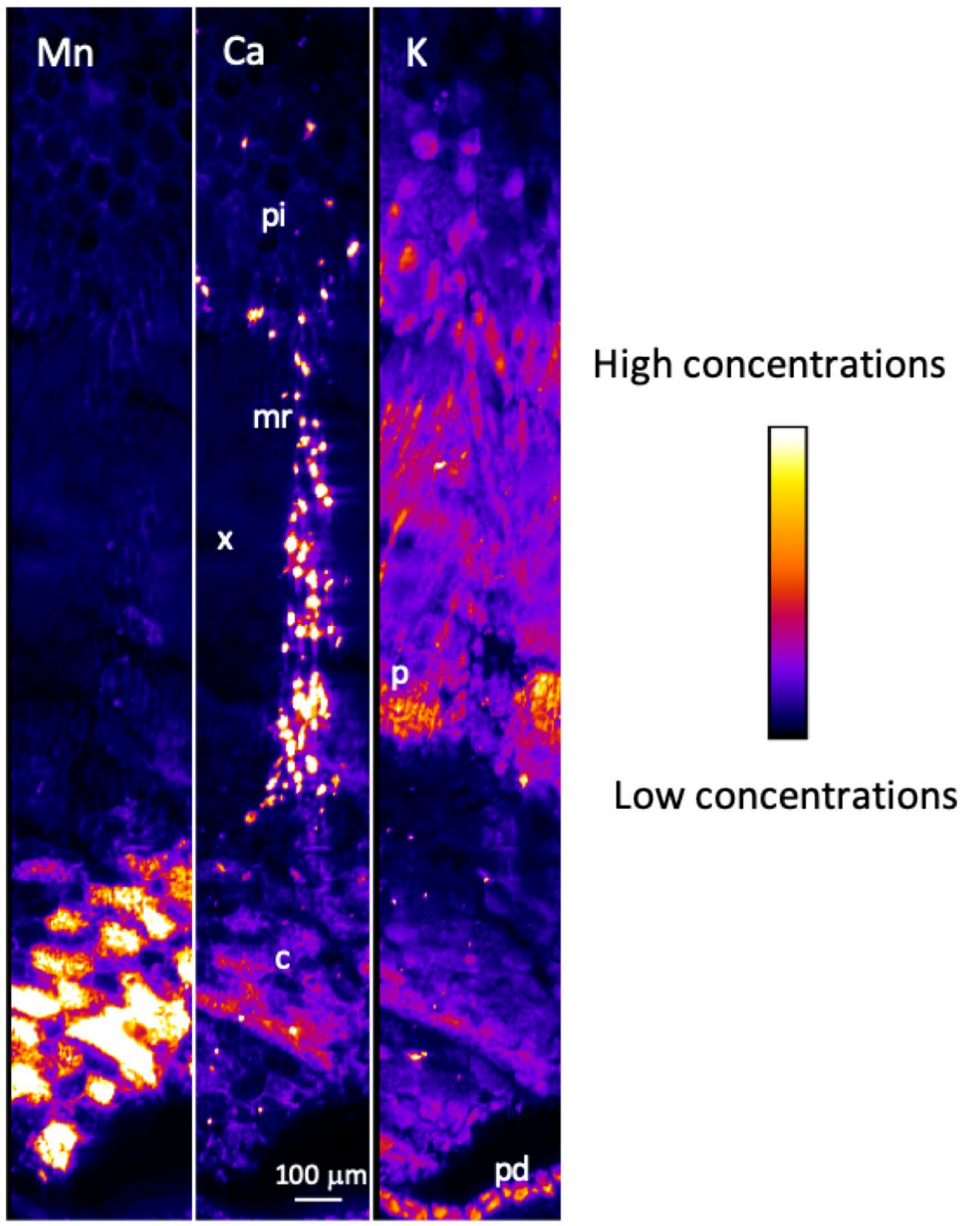
μXRF maps of Mn, Ca and K μXRF for a cross-section of a frozen hydrated stem of transplanted *Grevillea meisneri*. The pixel size is 3 μm. The intensity scales are different between elements. *c* cortex, *mr* medullary ray, *p* phloem, *pi* pith, *pd* periderm, *x* xylem.

μXRF maps of the stem showed that Mn was highly concentrated in the cortex, sixfold higher than in other parts of the stem, and was also found in the vascular tissues, particularly in the phloem (Fig. 4 and Figs. S5, S6). Ca was also localized in the cortex and highly concentrated in cells aligned in axial rays around the xylem. A similar Ca enrichment in medullary rays was previously reported in the Ni-hyperaccumulator *Rinorea* cf. *javanica*^96^. A similar distribution was observed for S and Mg. K was homogeneously distributed through the stem, with a slightly higher concentration in the phloem, whereas P attained its highest concentrations in the xylem. Cl was mostly in the cortex as well as in the inner periderm.

**Figure 5:**
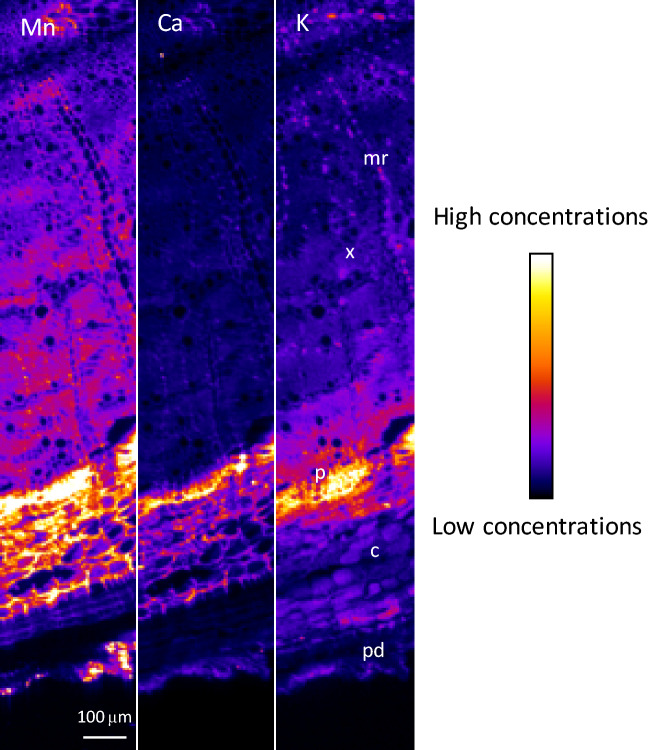
μXRF maps of Mn, Ca and K for a cross-section of a frozen hydrated primary root of transplanted *Grevillea* *meisneri*. The pixel size is 3 μm. The intensity scales are different between elements. *c* cortex,* mr* medullary ray, *p* phloem, *pd* periderm, *x* xylem.

μXRF maps of the primary roots showed that Mn was mainly localised in the cortex and the phloem, the cortex showing the highest Mn concentration (Fig. 5 and Figs. S7, S8). In the cortex, Mn was mostly localized in the apoplastic spaces. Similarly to Mn, Ca was located in the cortex and in the phloem, but with the highest concentration in the latter tissue. Cl was concentrated in the phloem and inner periderm. K distribution mirrored that of Cl with a strong enrichment in the phloem. P was concentrated in the vascular tissues of the roots.

**Figure 6:**
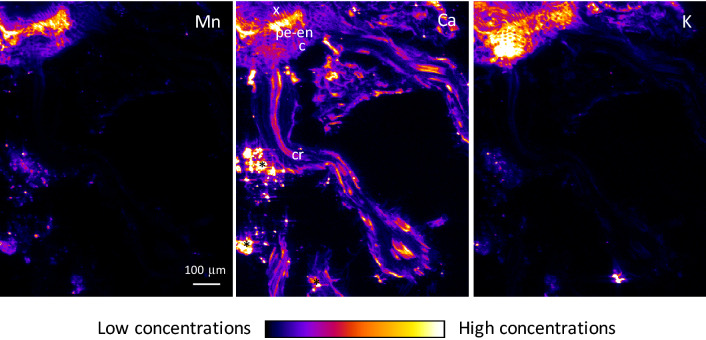
μXRF maps of Mn, Ca and K for a cross-section of frozen hydrated cluster root of transplanted *Grevillea* *meisneri*. The pixel size is 3 μm. The intensity scales are different between elements. *c* cortex, *cr* cluster rootlet, *p* phloem, *pe–en*, pericycle–endodermis, *x* xylem. Asterisks indicate soil particles.

μXRF maps of the cluster roots showed that Mn was mainly concentrated in the phloem and in the pericycle–endodermis layers of the parent cluster root (Fig. 6 and Figs. S9–S11). Mn was found in lower concentration in the cortex. Ca was distributed throughout the parent cluster root and the rootlet, principally in the pericycle–endodermis layers and phloem. Cl was concentrated in the cortex in the parent cluster roots, and K, Mg and S were evenly distributed throughout the parent cluster roots, with a minor enrichment in the phloem. P was mainly localized in the phloem of the parent cluster roots.

### Co-localization of elements

An example of the graphs depicting the correlation circles between the first two principal components of the PCA of the lower and upper epidermis of the leaf margin of *G. meisneri* was presented in Fig. S12. Interestingly, in each organ a strong positive correlation between Mn and Ca concentrations was observed (*P* ≤ 0.0001, Table 3 and Table S8). Overall, no strong positive correlations across all tissues were found between Mn and any other physiological element. Mn and Mg were both concentrated in the upper epidermis of the leaf and in the cortex of the stem, and both K and Cl showed positive correlations with Mn in the phloem of the parent cluster root.

**Table 3:** Pearson correlation coefficients between Mn and other physiological elements among the main Mn sinks of the different organs of *Grevillea meisneri* (*ue* upper epidermis, *le* lower epidermis, *c* cortex, *pe–en* pericycle–endodermis layers, *p* phloem). P-values are all ≤ 0.0001, except where otherwise indicated. Values of Pearson correlation coefficient higher than 0.5 are indicated in bold characters.

Organ	Tissue	Mn/Ca	Mn/Cl	Mn/K	Mn/Mg	Mn/P	Mn/S
Leaf margin	ue	**0.68**	**0.53**	0.40	0.47	0.40	**0.51**
	le	**0.94**	0.48	0.45	0.30	0.43	0.44
Leaf mid-rib	ue	**0.98**	0.28	0.45	**0.73**	0.39	0.45
	le	**0.73**	0.23	0.27	0.36	0.32	0.30
Stem	c	**0.60**	0.11	0.34	**0.56**	0.47	0.41
Primary root	c	**0.89**	0.48	0.38	0.28	0.26	0.12
Parent cluster Root	pe–en	**0.84**	0.43	0.49	0.45	0.46	0.45
	p	**0.94**	**0.72**	**0.73**	0.25	− 0.11^#^	0.04^##^

^#^*P*-value: 0.0007.

^##^*P*-value: 0.17.

### µXANES spectra

Based on the µXRF elemental maps obtained for the leaves of transplanted *G. meisneri*, µXANES spectra were collected on points of interest chosen for different concentrations in Mn and/or Ca, meaning lower epidermis (richer in Mn than in Ca), upper epidermis (richer in Ca than in Mn), spongy mesophyll, palisade mesophyll and vascular bundles (each ones with poor concentrations of Mn and Ca) (Fig. S13). The aim was to study the potential spatial heterogeneity for Mn speciation all along the epidermal layers and the mesophyll of *G. meisneri*’ leaves.

For every tissue, the normalized XANES spectra were strongly characteristic of Mn(II), by both edge energies and shapes of the spectra. The pre-edge peak at 6540 eV, the intense white-line peak at 6553 eV and the spectral shape on the high-energy side of the white-line peak around 6566 eV are indicative of an octahedral or pseudo-octahedral coordinative environment around Mn(II)^79,97^. No differences were observed with the variation of concentration of Mn, of Ca nor the ratio Mn/Ca.

## Discussion

Micro-X-ray elemental mapping techniques give in situ information on the cellular distribution of elements within plant tissues, effectively immobilizing their metabolic processes to yield material whose state is as close as possible to the native state of the sample. It preserves not only the morphological structures of the sample but also the in vivo distribution and chemical form of the cellular elements. Indeed, the fast freezing of freshly excised small samples, as followed in this study, avoids the formation of ice crystals which would lead to artefacts, such as damage to cell structure, elemental redistribution or loss of cell solutes^90,98^. The use of Synchrotron radiation micro-X-Ray Fluorescence spectroscopy (μXRF) enabled mapping of Mn together with Ca, Cl, K, Mg, P and S, in the different tissues of *G. meisneri*.

For both transplanted and spontaneously growing plants, evaluation of tissue-Mn in across all organs of *G. meisneri* showed that its leaves contain highest concentrations, as expected for a (hyper)accumulated metal. Even higher concentrations of Mn could have been expected in leaves, as a mean concentration of 0.7 wt % (and a maximum of 1.1 wt %) was previously found in leaves of *G. meisneri*^7^. However, the lower values for Mn concentrations obtained in this study, which used young leaves, could be explained by the previously established relationship between leaf age and foliar concentration of Mn, the oldest leaves having the highest Mn concentrations.

Substantial concentrations of Mn were also found in roots. Interestingly, no Ni was detected using MP-AES in any part of the plant, although *G. meisneri* grew on Ni-enriched soil and was surrounded by Ni-hyperaccumulator species. Specific transporters of Mn^2+^, such as carboxylate complexes that are secreted by cluster roots of Proteaceae^99^, might explain this specific uptake mechanism towards Mn versus Ni. µXANES spectra obtained in many points of every tissue of *G. meisneri* demonstrated the predominance of Mn(II) oxidation state, which was consistent with previous investigations on the speciation of Mn in plants^46^. Some studies have suggested oxalate as binding ion to coordinate soluble foliar Mn(II)^39,100–102^. Foliar Mn speciation in various Mn-(hyper)accumulating plants, including *G. exul*, was extensively studied by XANES and EXAFS by Fernando et al.^79^. It was demonstrated that citrate and malate were the most plausible counter-ions associated to Mn(II), with some evidence in favor of malate. By comparison with the data obtained by Fernando et al., the µXANES spectra collected in the leaves of *G. meisneri* clearly supported O-donor ligands as counter-ions to Mn(II), and mostly citrate and malate. Similarly, µXANES data alone cannot differentiate precisely which carboxylate is involved in the binding of Mn(II). Ion chromatography analyses of leaves of *G. meisneri* revealed high contents of malate and oxalate, while citrate could not be detected. These analyses supported that malate could be involved in the sequestration of Mn in leaves of *G. meisneri* while high oxalate content could come from Ca-oxalate crystals observed in the leaf mesophyll. Crystalline Ca-oxalate had already been reported in many previously studied Mn-(hyper)accumulators^73,103^ as well as in *Grevillea exul* ssp *rubiginosa* seeds^104^. The accumulation of Ca in the leaves of *G. meisneri* in sufficient quantity to form calcium oxalate crystals could signify some adaptation of the plants for the uptake of large amounts of Ca from ultramafic soils characterized by particularly low exchangeable Ca content^105^.

In leaves of *G. meisneri*, Mn was mainly sequestered in the epidermal tissues. The foliar sequestration of metals, including Mn^65–69^, Ni^59,61–64^, Zn and Cd^58,60^, usually occurs in non-photosynthetic tissues, enabling avoidance of any damage to cell metabolic activities. Here, the asymmetrical accumulation of Mn in epidermal layers might act as a chemical defense strategy against herbivory. In favour of this hypothesis is the observation that the lower epidermis presented more enrichment in Mn than the upper one, similar to findings for other heavy metals in other accumulator plants^60,61,106–108^. Many insect herbivores prefer to feed on leaf undersides, where the cuticle is often less tough, and where the insects have a little bit of shade. Although Mn is considered less toxic to plants than other metals, its detoxification is needed for Mn-hyperaccumulators and their strategy relies on cell vacuolation for Mn sequestration in leaves^60,73,109^. Although the resolution of μXRF was not sufficient to discern subcellular elemental localizations, the shape of Mn distribution in the epidermal layers, and the wide vacuolar volumes of the large dermal cells compared to the thin cells of the palisade, strongly suggest a vacuolar sequestration, without excluding localization in cell walls.

In stems, the presence of Mn in the vascular tissues and in the cortex could be respectively explained by its transportation from root to shoot and from shoot to older leaves^56^, and by its sequestration. Both mechanisms could represent an adaptive strategy developed by *G. meisneri* facing excessive concentrations of Mn.

Mn distribution in the primary roots revealed the early uptake of Mn through the apoplastic pathway in the cortex, before entering the symplast in the endoderm, then reaching the phloem, but not the xylem. Similar distribution of Mn has also been observed in the roots of *Gossia fragrantissima* (Myrtaceae)^110^. Further investigations are needed to deepen our understanding of Mn transportation, its low mobility in the phloem sap^111^ and its transportation from root to shoot in the xylem^112^, mainly considering that these processes generally observed in plants are not entirely followed by some Australian Gossia Mn-hyperaccumulator species, *G. grayi* and *G. shepherdii*^113^.

Strong positive Mn/Ca correlations were observed in every tissue of *G. meisneri* where Mn was the most concentrated. Such positive Mn/Ca correlations have also been found in the dermal layers of other Mn-hyperaccumulators, *Garcinia amplexicaulis* (Clusiaceae)^73^, *Gossia grayi* and *G. shepherdii* (Myrtaceae)^74^. The co-localization of Ca and Mn in the same tissues of *G. meisneri* might in some way facilitate the formation of mixed Ca–Mn oxides upon preparation of Eco-CaMnOx ecocatalysts from this plant. The original structure and reactivity of Eco-CaMnOx are inherently linked to the leaves from which they come from: this represents the peculiar vegetal footprint of ecocatalysts. It might also indicate shared transport systems that could facilitate the formation of mixed oxides as well. Interestingly, the distributions of these two metal elements are negatively correlated in the mesophyll of two other Mn-hyperaccumulating *Gossia* species, the main Mn sequestration site for these species^74^. If co-localization of the two elements facilitates formation of the mixed Mn–Ca oxide, this would lead to the predictions that (i) mixed Mn–Ca oxides would be absent from, or present in much lower concentrations in, ecocatalysts prepared from these *Gossia* species; and (ii) there is no shared transport system for Mn and Ca in these *Gossia* species. Unfortunately, whether such oxides occur in ecocatalysts prepared from any *Gossia* species appears still to be unknown. Whether shared Mn/Ca transport systems exist is not known for any Mn-hyperaccumulator. Mn(II) is the predominant ionic form of Mn in plants and shares similar radius and chemical features (hardness according to HSAB theory) with Ca(II)^114^. This similarity could explain the positive Mn/Ca correlation via a cooperative uptake and transportation pathway in *G. meisneri*^115^. Indeed, many transporters of Ca(II), such as some membrane Ca^2+^ channels, are reported to be permeable to Mn in *Arabidopsis thaliana* (Brassicaceae) and in *Acanthopanax sciadophylloides* (Araliaceae)^116–120^.

In efforts to revegetate metal-bearing mine waste sites, direct seeding is almost universally used as a seeding strategy. In fact, the review by Tordoff et al. mentions transplantation only once^121^. However, in reforestation efforts in a diversity of contexts, studies have shown transplanted plants to have higher establishment, survivorship and growth rates than plants from direct seeding^122^. We showed that even in the harsh climatic and edaphic conditions in a site degraded by open-cast mining, nursery-grown transplants of a metal-tolerant native plant established successfully, accumulated Mn as efficiently as plants growing naturally in the sites, and sequestered Mn within their tissues in the same way as naturally growing plants.

In metalliferous mine waste sites, transplanting should confer even greater advantages relative to direct seeding than in other environments. The harsh, extreme conditions should make seed survival and seedling establishment particularly difficult. Bypassing the vulnerable seed-to-seedling life-history transition by growing young seedlings in favourable nursery environments could greatly increase the survivorship and growth rate of transplanted seedlings in mine waste sites. As in other parts of the world where naturally metalliferous soils occur, New Caledonia has native metal-tolerant plants that are highly suitable for revegetation efforts. Because seed supply of these native plants is often highly limited, seeding strategies based on transplanted nursery-grown seedlings will be much more efficient than direct seeding, in which a large proportion of seeds and young seedlings die before establishment.

Bermúdez-Contreras et al. pointed out a potential limitation of transplantation as a seeding strategy^123^. Establishment and growth of the young plant may depend on below-ground interactions, for example, the early establishment of associations with the locally adapted soil microflora (mycorrhizal fungi, but also other soil fungi and bacteria). With direct seeding, the young plant can form these associations, and benefit from them, at the start of its life. In contrast, nursery-reared seedlings are usually grown in tubes containing some standard soil, not the native soil of the sites to be revegetated. Transplanted to these sites, they may suffer a deficit of, or a lag in, colonization by the native microbiota. We circumvented potential problems caused by such disruption of underground interactions by growing nursery-reared seedlings in native soil of the site before mining. The native soil introduced with the transplants could help inoculate the site after removal of its topsoil. Our results suggest that transplantation should be increasingly adopted as a seeding strategy in restoration efforts in degraded mining sites.

## Supplementary Information

Supplementary Information.
