# Supplementary material for: Manganese distribution in the Mn-hyperaccumulator Grevillea meisneri from New Caledonia
Source: Sci Rep. 2021 Dec 10;11:23780. doi: 10.1038/s41598-021-03151-9 (PMC8664926; doi:10.1038/s41598-021-03151-9)
Supplement: Supplementary file 1 — Supplementary Information. [file 41598_2021_3151_MOESM1_ESM.docx]

**Manganese distribution in the Mn-hyperaccumulator *Grevillea meisneri* from New Caledonia**

Camille Bihanic,*^a^* Eddy Petit,*^b^* Roseline Perrot,*^c^* Lucie Cases,*^a^* Armelle Garcia,*^a^* Franck Pelissier,*^a^* Cyril Poullain,*^a^* Camille Rivard,*^d^* Martine Hossaert-McKey,*^a, e^* Claude Grison*^a*^*

*^a^* Laboratoire de Chimie Bio-inspirée et d’Innovations Écologiques, ChimEco, UMR 5021, CNRS – Université de Montpellier, Cap Delta, 1682 Rue de la Valsière, 34790 Grabels, France

*^b^* Institut Européen des Membranes, IEM – UMR 5635, ENSCM, CNRS – Université de Montpellier, 34090 Montpellier, France

*^c^* Service des Laboratoires Officiels Vétérinaires Agroalimentaires et Phytosanitaires de Nouvelle-Calédonie, DAVAR, New Caledonia, France

*^d^* Synchrotron SOLEIL, 91190 saint-Aubin, France

*^e^* TRANSFORM, UAR 1008, INRAE, 44316 Nantes, France

*^f^* CEFE, Université de Montpellier, CNRS, EPHE, IRD, 1919 route de Mende, 34293 Montpellier cedex 5, France

**Author for correspondence:**

Claude Grison

Tel: +33 7 86 85 44 54

Email: [claude.grison@cnrs.fr](mailto:claude.grison@cnrs.fr)

**SUPPLEMENTARY METHODS**

*Nursery growth conditions*

The germination of seeds was made in a mixture of river sand and coir. Round particles of river sand avoid abrasion on seedlings during removal. The sprouts were then transplanted into small pots with a mixture of topsoil (1/3), compost (1/3) and coir (1/3). No manganese was added during the plants’ growth in the nursery.

Plants were watered twice a day during 5 minutes in the wet season and twice a day during 10 minutes during the dry season. One month before plantation, watering was gradually decreased: seedlings were watered only once each day during the first two weeks, then once every two days for the last two weeks. The temperature during nursery growth was not regulated, but was the ambient temperature, which ranged from about 30 °C (day) to 20 °CC (night) in the wet season and 35 °C to 25 °C in the dry season.

Monitoring of the plants’ growth

Survival and size of each transplanted plant, and of plants naturally developing *in situ* near the transplanted plants, was monitored once a year in the studied mining site. Two parameters, height of the plant and root collar diameter, were chosen as measures of plant size. Both can be quickly and easily measured in a reproducible manner and provide useful information on the response of plants to environment.

Two other parameters were monitored to study the efficiency of the phytoremediation program: the biomass production and the foliar mineral composition. The biomass production was estimated by counting the number of leaves and measuring the minimal and maximal dimensions of each plant. In order to minimize any impact of sampling on the plant’s growth, only a few leaves were sampled for analyses of mineral composition.

SUPPLEMENTARY FIGURES AND TABLES

**Table S1.** Composition and chemical characteristics of soils of the 16 parcels of ‘Creek à Paul’ under rehabilitation, established by ICP-MS analyses.

| **Site** | **pH** | | **Available N**  **(mg/kg)** | | **Available P (mg/kg)** | **Extrait DTPA (mg/kg)** | | **Elemental composition (mg/kg)** | | | | | | | | | | | |
| --- | --- | --- | --- | --- | --- | --- | --- | --- | --- | --- | --- | --- | --- | --- | --- | --- | --- | --- | --- |
|  | **H_2_O** | **KCl** | **N(NO_3_)** | **N(NH_4_)** |  | **Mn** | **Ni** | **Ca** | **Mg** | **Na** | **K** | **Fe** | **Co** | **Cr** | **Mn** | **Ni** | **Al** | **P** | **Zn** |
| **CAP A** | 7.07 | 5.51 | 0.1 | 8.9 | 1 | 604 | 170 | 7 196 | 56 506 | 2 389 | 710 | 127 476 | 1 204 | 12 119 | 8 479 | 6 104 | 36 082 | 13 | 124 |
| **CAP B** | 7.00 | 5.19 | 0.2 | 4.8 | 1 | 424 | 135 | 187 | 27 526 | 458 | 118 | 120 489 | 699 | 7 113 | 1 240 | 15 316 | 13 692 | 1 | 93 |
| **CAP C** | 7.08 | 5.47 | 0.0 | 7.2 | 0 | 492 | 131 | 1 148 | 32 094 | 859 | 106 | 123 277 | 724 | 14 177 | 4 178 | 7 053 | 24 080 | 14 | 133 |
| **CAP D** | 6.98 | 5.46 | 0.1 | 7.2 | 0 | 343 | 144 | 5 458 | 72 842 | 2 506 | 303 | 126 155 | 1 447 | 13 019 | 9 333 | 5 600 | 41 703 | 4 | 120 |
| **CAP E** | 6.33 | 4.69 | 0.2 | 5.9 | 0 | 416 | 216 | 9 699 | 57 289 | 2 934 | 331 | 120 178 | 1 182 | 12 902 | 8 667 | 4 723 | 44 143 | 12 | 119 |
| **CAF F** | 6.44 | 4.82 | 0.3 | 7.4 | 0 | 531 | 138 | 446 | 28 481 | 628 | 104 | 142 882 | 1 264 | 20 883 | 6 915 | 5 638 | 31 025 | 22 | 176 |
| **CAP G** | 6.31 | 4.95 | 1.0 | 7.1 | 0 | 331 | 200 | 701 | 34 510 | 657 | 151 | 158 153 | 1 135 | 17 846 | 8 167 | 9 455 | 29 295 | 24 | 147 |
| **CAP H** | 6.74 | 4.56 | 0.2 | 4.3 | 0 | 227 | 202 | 1 305 | 59 585 | 742 | 113 | 122 635 | 381 | 11 871 | 2 649 | 12 152 | 21 866 | 16 | 111 |
| **CAP I** | 6.89 | 4.42 | 0.6 | 3.7 | 2 | 231 | 232 | 2 401 | 68 530 | 660 | 164 | 119 412 | 475 | 12 267 | 3 384 | 12 132 | 21 213 | 1 | 128 |
| **CAP J** | 6.84 | 5.27 | 0.2 | 4.8 | 2 | 255 | 201 | 1446 | 46987 | 859 | 96 | 101863 | 526 | 8946 | 3052 | 9142 | 19419 | 5 | 162 |
| **CAP K** | 6.58 | 5.02 | 0.4 | 4.1 | 1 | 167 | 233 | 1504 | 49610 | 891 | 95 | 110301 | 449 | 11182 | 2015 | 10325 | 22204 | 14 | 180 |
| **CAP L** | 6.51 | 4.89 | 0.7 | 5.7 | 2 | 342 | 189 | 885 | 35757 | 592 | 56 | 109096 | 560 | 11867 | 2877 | 7856 | 22647 | 21 | 160 |
| **CAP M** | 5.95 | 4.48 | 0.3 | 9.4 | 2 | 163 | 185 | 713 | 28522 | 460 | 125 | 101200 | 395 | 7397 | 1749 | 7576 | 13700 | 4 | 140 |
| **CAP N** | 6.59 | 4.90 | 0.5 | 5.9 | 1 | 509 | 144 | 744 | 26779 | 634 | 88 | 99035 | 555 | 16125 | 2794 | 4740 | 24822 | 14 | 166 |
| **CAP O** | 6.05 | 4.48 | 0.6 | 5.6 | 0 | 370 | 189 | 829 | 26714 | 761 | 70 | 99570 | 572 | 11839 | 2885 | 6948 | 22860 | 9 | 162 |
| **CAP P** | 6.29 | 4.76 | 0.5 | 7.8 | 1 | 501 | 192 | 1154 | 29977 | 1267 | 103 | 115313 | 1225 | 14077 | 7480 | 6886 | 24119 | 5 | 174 |

**Table S2.** Composition and quantity of fertilizers applied to transplanted G. meisneri seedlings at ‘Creek à Paul’.^a^

|  | **Quantity (g/plant)** | **N (kg/ha)** | **P (kg/ha)** | **K (kg/ha)** |
| --- | --- | --- | --- | --- |
| **Yates nutricote** | 13.85 | 200 | 88 | 166 |
| **Superphosphate triple** | 8.8 | 0 | 200 | 0 |
| **Total** | 22.65 | 200 | 288 | 166 |

*^a^* The quantity of fertilizer added was adjusted in order to have the same amount of nitrogen provided for the different treatments on the various mining sites under rehabilitation by our group.

**Table S3.** Analysis of Yates NUTRICOTE fertilizer provided by the supplier.

| **Yates NUTRICOTE** | **Composition (%w/w)** |
| --- | --- |
| Nitrogen (N) as nitrate | 7.0 |
| Nitrogen (N) as ammonium | 7.0 |
| **Total Nitrogen** | **14.0** |
|  |  |
| Phosphorus (P) as water soluble | 5.2 |
| Phosphorus (P) as citrate soluble | 0.9 |
| **Total Phosphorus** | **6.1** |
|  |  |
| **Total Potassium (K) as nitrate** | **11.6** |
| Calcium (Ca) as phosphates | 4.0 |
| Inert coating | 5.7 |

**Table S4.** Analysis of Superphosphate fertilizer provided by the supplier.

| **Superphosphate** | **Composition (%w/w)** |
| --- | --- |
| **Total Nitrogen** | **0** |
|  |  |
| P_2_O_5_ | 46 |
| **Total phosphorus** | **20** |
|  |  |
| K_2_O | 0 |
| CaO | 15 |
| S | 1,3 |

**Table S5.** Foliar elemental composition of G. rosa ssp. jenkinsii, determined by MP-AES analyses.

|  | **Composition (ppm (%RSD))** | | | | |
| --- | --- | --- | --- | --- | --- |
| Leaves of *‘G. rosa* J*enkinsii’* | **Mn** | **Ca** | **K** | **Mg** | **Na** |
|  | 920 (1,32) | 17640 (1,21) | 4051 (0,82) | 2390 (2,60) | 122 (2,77) |

**Table S6.** Foliar P and S composition of various species of Grevillea genus, determined by ICP-MS.

|  | **Composition (ppm (%RSD))** | |
| --- | --- | --- |
|  | **P** | **S** |
| *Grevillea meisneri* leaves | 228.79 (2.15) | 1294.50 (6.48) |
| *Grevillea gillivrayi* leaves | 240.56 (2.35) | 1816.94 (11.23) |
| *Grevillea exul* ssp. *exul* leaves | 282.99 (1.26) | 2561.52 (15.03) |
| *Grevillea ‘rosa Jenkinsii’* leaves | 1183.58 (0.83) | 2232.81 (8.00) |

**Table S7.** Concentrations of anions in leaves of Grevillea meisneri, determined by ion chromatography analyses.

| **Anions** | **Malate** | **Oxalate** | **Citrate** | **SO_4_^2-^** | **PO_4_^3-^** |
| --- | --- | --- | --- | --- | --- |
| **Concentration (mg.L^-1^)** | 14.6 | 7.7 | nd | 10.9 | 39.2 |

*nd: not detected.*

**Figure S1.** Cl, Mg, P and S μXRF maps of a frozen hydrated leaf margin cross-section of transplanted G. meisneri. The pixel size is 3 μm. The intensity scales are different between elements. c, cuticle; le, lower epidermis; pm, palisade mesophyll; sm, spongy mesophyll, ue, upper mesophyll.

**Figure S2.** Mn, Ca, Cl, K, Mg, P and S μXRF maps of a frozen hydrated leaf mid-rib cross-section of transplanted G. meisneri. The pixel size is 3 μm. The intensity scales are different between elements. le, lower epidermis; pm, palisade mesophyll; sm, spongy mesophyll; ue, upper epidermis; vb, vascular bundles.

S


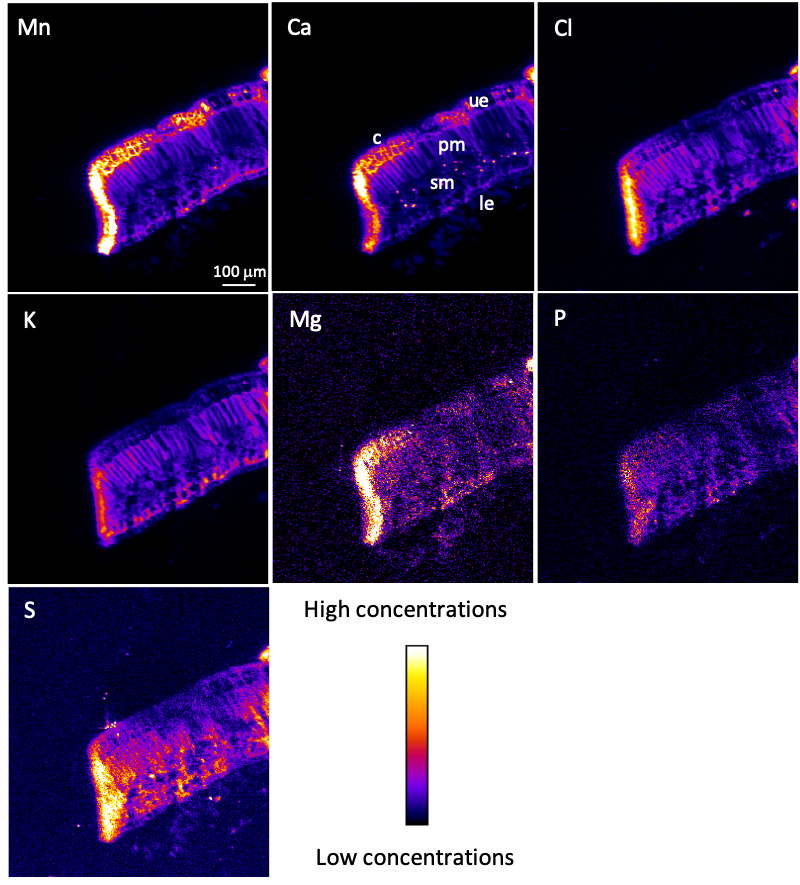


**Figure S3.** Mn, Ca, Cl, K, Mg, P and S μXRF maps of a frozen hydrated leaf margin cross-section of G. meisneri spontaneously growing in the site. The pixel size is 3 μm. The intensity scales are different between elements. c, cuticle; le, lower epidermis; pm, palisade mesophyll; sm, spongy mesophyll, ue, upper mesophyll.


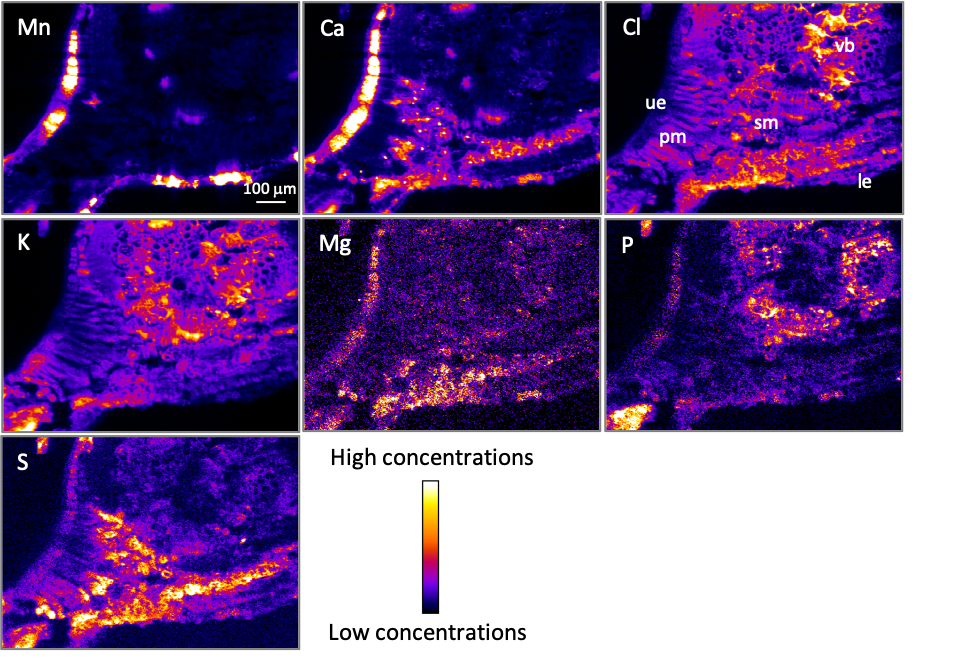


**Figure S4.** Mn, Ca, Cl, K, Mg, P and S μXRF maps of a frozen hydrated leaf mid-rib cross-section of G. meisneri spontaneously growing in the site. The pixel size is 3 μm. The intensity scales are different between elements. le, lower epidermis; pm, palisade mesophyll; sm, spongy mesophyll; ue, upper epidermis; vb, vascular bundles.


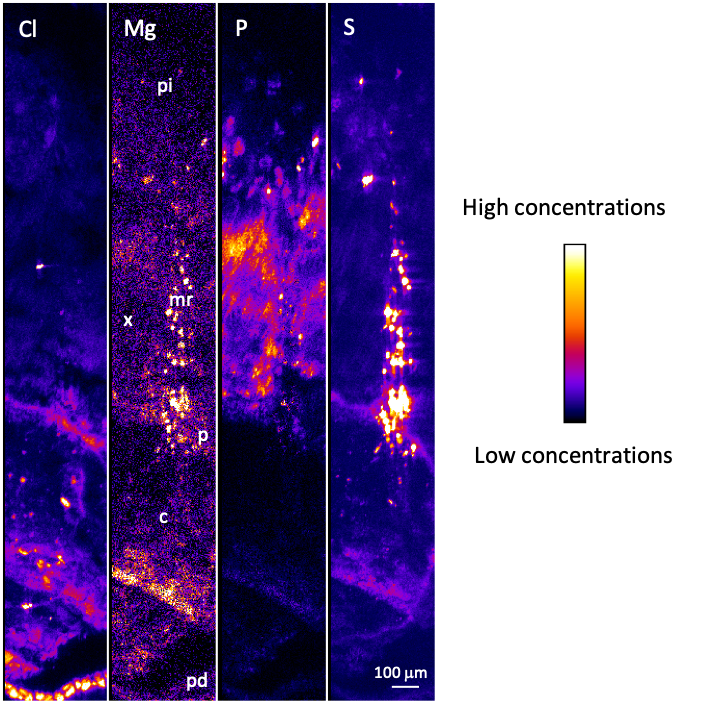


**Figure S5.** Cl, Mg, P and S μXRF maps of a frozen hydrated stem cross-section of transplanted G. meisneri. The pixel size is 3 μm. The intensity scales are different between elements. c, cortex; mr, medullary ray; p, phloem; pi, pith; pd, periderm; x, xylem.


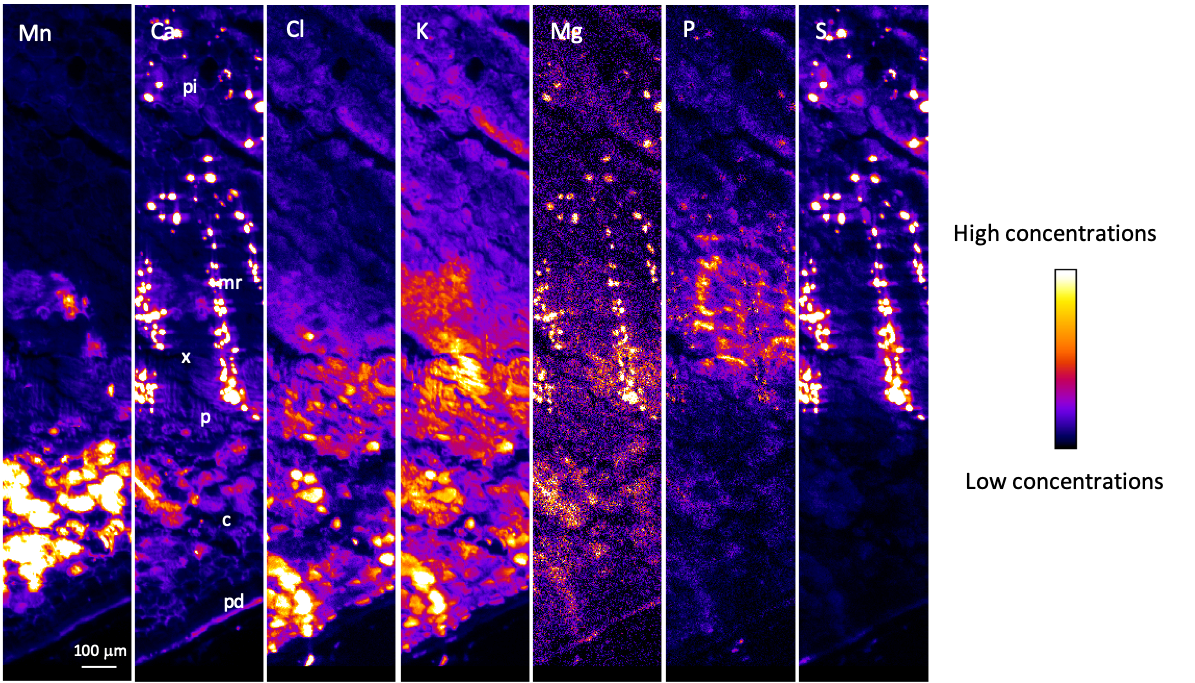


**Figure S6.** Mn, Ca, Cl, K, Mg, P and S μXRF maps of a frozen hydrated stem cross-section of G. meisneri spontaneously growing in the site. The pixel size is 3 μm. The intensity scales are different between elements. le, lower epidermis; pm, palisade mesophyll; sm, spongy mesophyll; ue, upper epidermis; vb, vascular bundles.

**Figure S7.** Cl, Mg, P and S μXRF maps of a frozen hydrated primary root cross-section of transplanted G. meisneri. The pixel size is 3 μm. The intensity scales are different between elements. c, cortex; mr, medullary ray; p, phloem; pd, periderm; x, xylem.


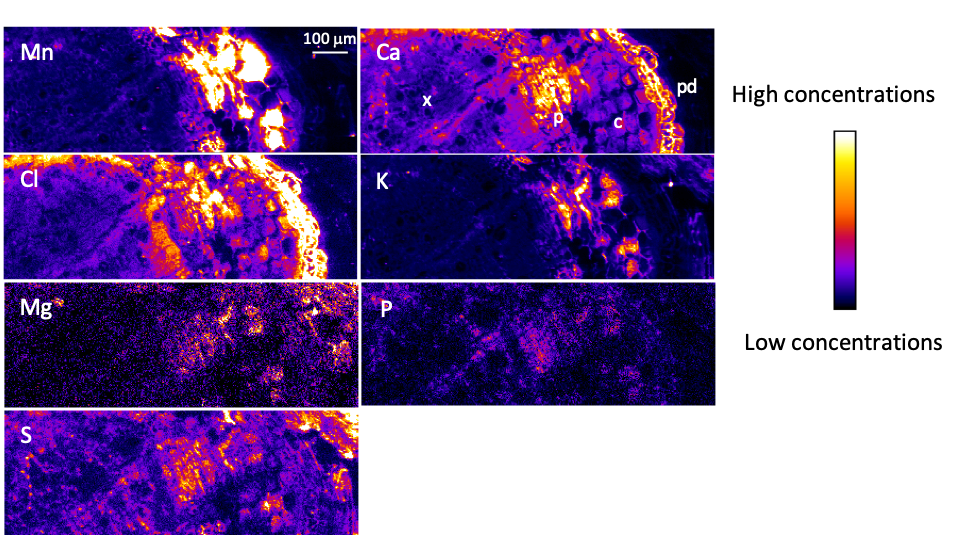


**Figure S8.** Mn, Ca, Cl, K, Mg, P and S μXRF maps of a frozen hydrated primary root cross-section of G. meisneri spontaneously growing in the site. The pixel size is 3 μm. The intensity scales are different between elements. c, cortex; p, phloem; pd, periderm; x, xylem.

**

**Figure S9.** Mn, Ca, Cl, K, Mg, P and S μXRF maps of a frozen hydrated young root cross-section of transplanted G. meisneri. The pixel size is 3 μm. The intensity scales are different between elements. c, cortex; e, epidermis; p, phloem; pe-en, pericycle-endodermis; x, xylem.

**Figure S10.** Mn, K, Cl, Mg, P, S and Cr μXRF maps of a frozen hydrated cluster root cross-section of transplanted G. meisneri. The pixel size is 3 μm. The intensity scales are different between elements. Pixel intensities on Mn and K maps were over-increased to observed cluster rootlets. c, cortex; cr, cluster rootlet; p, phloem; pe-en, pericycle-endodermis; x, xylem. Asterisks indicate soil particles.


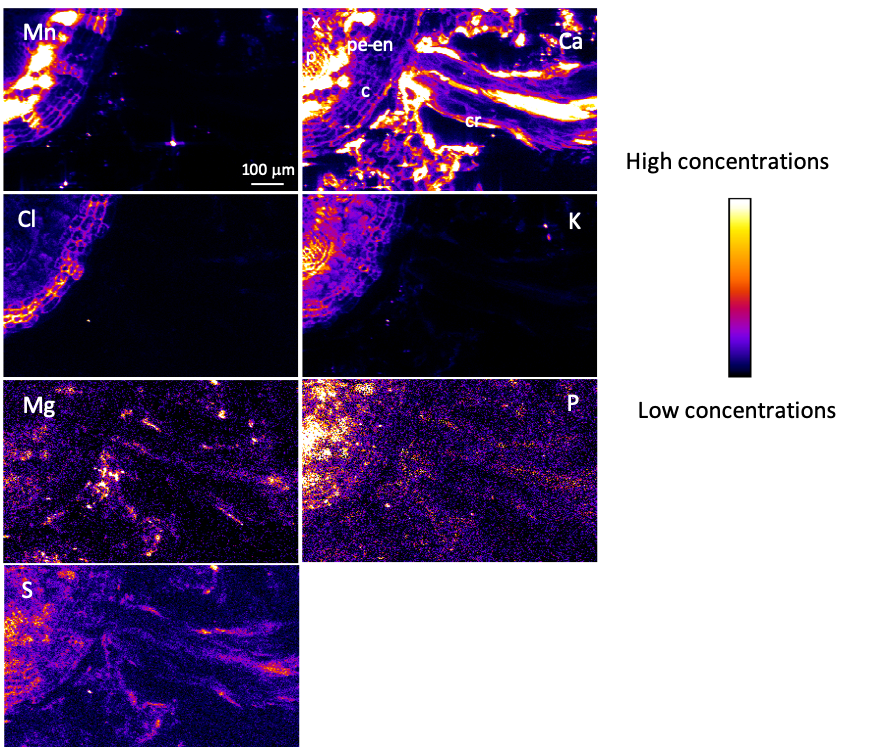


**Figure S11.** Mn, Ca, Cl, K, Mg, P and S μXRF maps of a frozen hydrated cluster root cross-section of G. meisneri spontaneously growing in the site. The pixel size is 3 μm. The intensity scales are different between elements. c, cortex; cr, cluster rootlet; p, phloem; pe-en, pericycle-endodermis; x, xylem.

**Figure S12**. Graph depicting the position of all the pixels of fluorescence intensity, for all the elements from the a) lower epidermis and b) upper epidermis, as well as the correlation circle between the first two principal components of the PCA of the c) lower epidermis and d) upper epidermis of the leaf margin cross section of G. meisneri.

**Table S8.** Correlation coefficients between physiological elements among the main Mn sinks of the different organs of G. meisneri (ue: upper epidermis; le: lower epidermis; c: cortex; pe-en: pericycle and endodermis layers; p: phloem). Correlation P-values of correlation coefficients are all ≤ 0.0001 except where noted otherwise. The most significant correlations are put in bold type (< 0.5).

|  | **Leaf margin** | | **Leaf mid-rib** | | **Stem** | **Primary root** | **Cluster parent root** | |
| --- | --- | --- | --- | --- | --- | --- | --- | --- |
|  | **ue** | **le** | **ue** | **le** | **c** | **c** | **pe-en** | **p** |
| **Ca/Cl** | **0.72** | **0.59** | 0.36 | 0.49 | 0.29 | 0.45 | 0.44 | **0.83** |
| **Ca/K** | 0.48 | **0.63** | **0.51** | **0.53** | 0.42 | 0.41 | **0.59** | **0.83** |
| **Ca/Mg** | **0.68** | 0.36 | **0.76** | 0.45 | **0.53** | 0.35 | **0.61** | 0.36 |
| **Ca/P** | **0.69** | 0.48 | 0.40 | 0.42 | 0.42 | 0.32 | **0.62** | 0.06# |
| **Ca/S** | **0.69** | **0.54** | 0.49 | **0.53** | **0.65** | 0.47 | **0.57** | 0.22 |
| **Cl/K** | **0.68** | **0.69** | **0.90** | **0.90** | **0.77** | **0.81** | **0.70** | **0.90** |
| **Cl/Mg** | **0.56** | 0.46 | 0.38 | 0.41 | 0.24 | 0.34 | 0.47 | **0.55** |
| **Cl/P** | **0.70** | **0.70** | 0.47 | **0.59** | 0.28 | 0.42 | 0.44 | 0.38 |
| **Cl/S** | **0.85** | **0.79** | **0.68** | **0.75** | 0.33 | 0.20 | **0.55** | **0.52** |
| **K/Mg** | 0.42 | 0.29 | 0.49 | 0.43 | 0.39 | 0.34 | 0.49 | **0.54** |
| **K/P** | 0.28 | 0.44 | **0.54** | **0.62** | 0.41 | **0.50** | **0.68** | 0.45 |
| **K/S** | **0.64** | **0.60** | **0.58** | **0.78** | 0.46 | 0.20 | **0.78** | **0.55** |
| **Mg/P** | **0.50** | **0.52** | 0.37 | 0.34 | 0.40 | 0.26 | **0.64** | 0.47 |
| **Mg/S** | **0.55** | **0.52** | 0.46 | 0.45 | 0.44 | 0.21 | **0.63** | 0.48 |
| **P/S** | **0.67** | **0.78** | 0.40 | **0.59** | 0.39 | 0.20 | **0.75** | **0.80** |

# *P*-value: 0.04


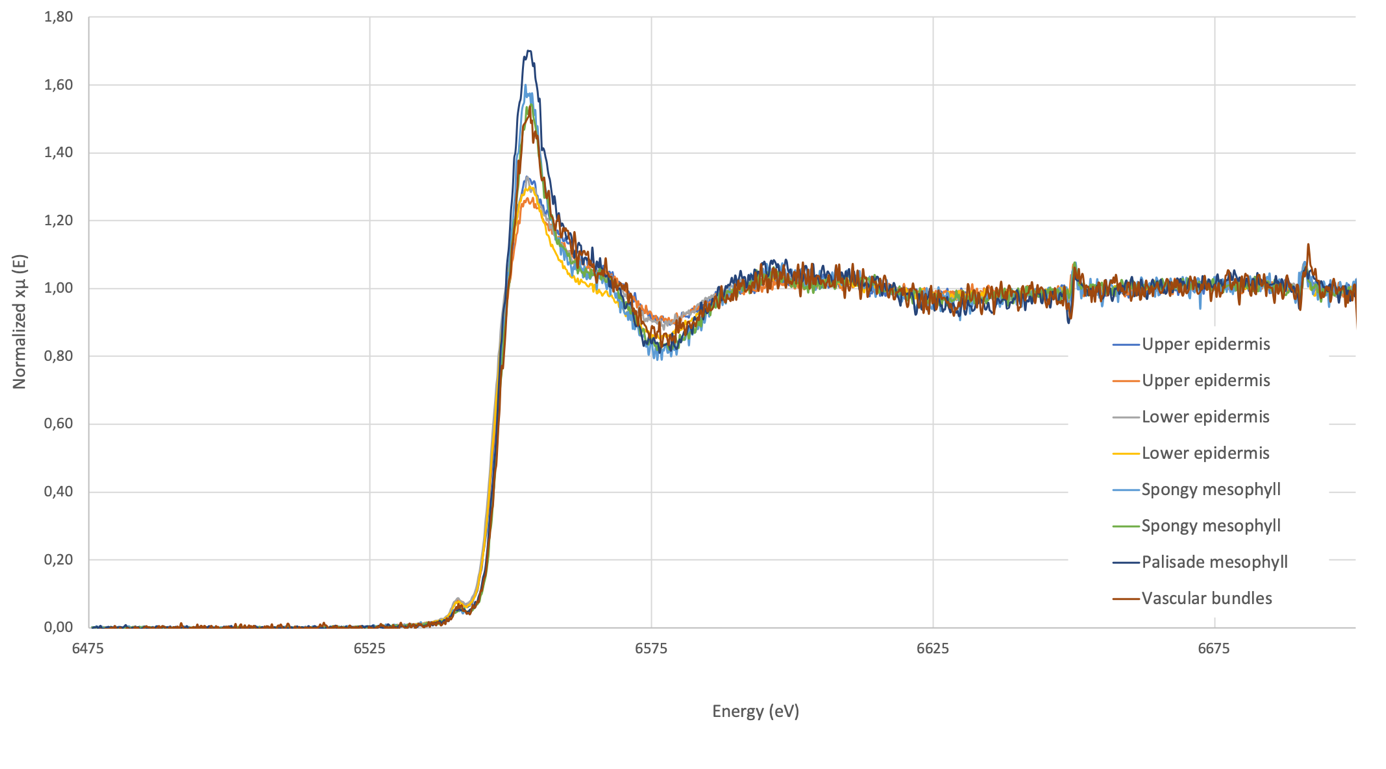

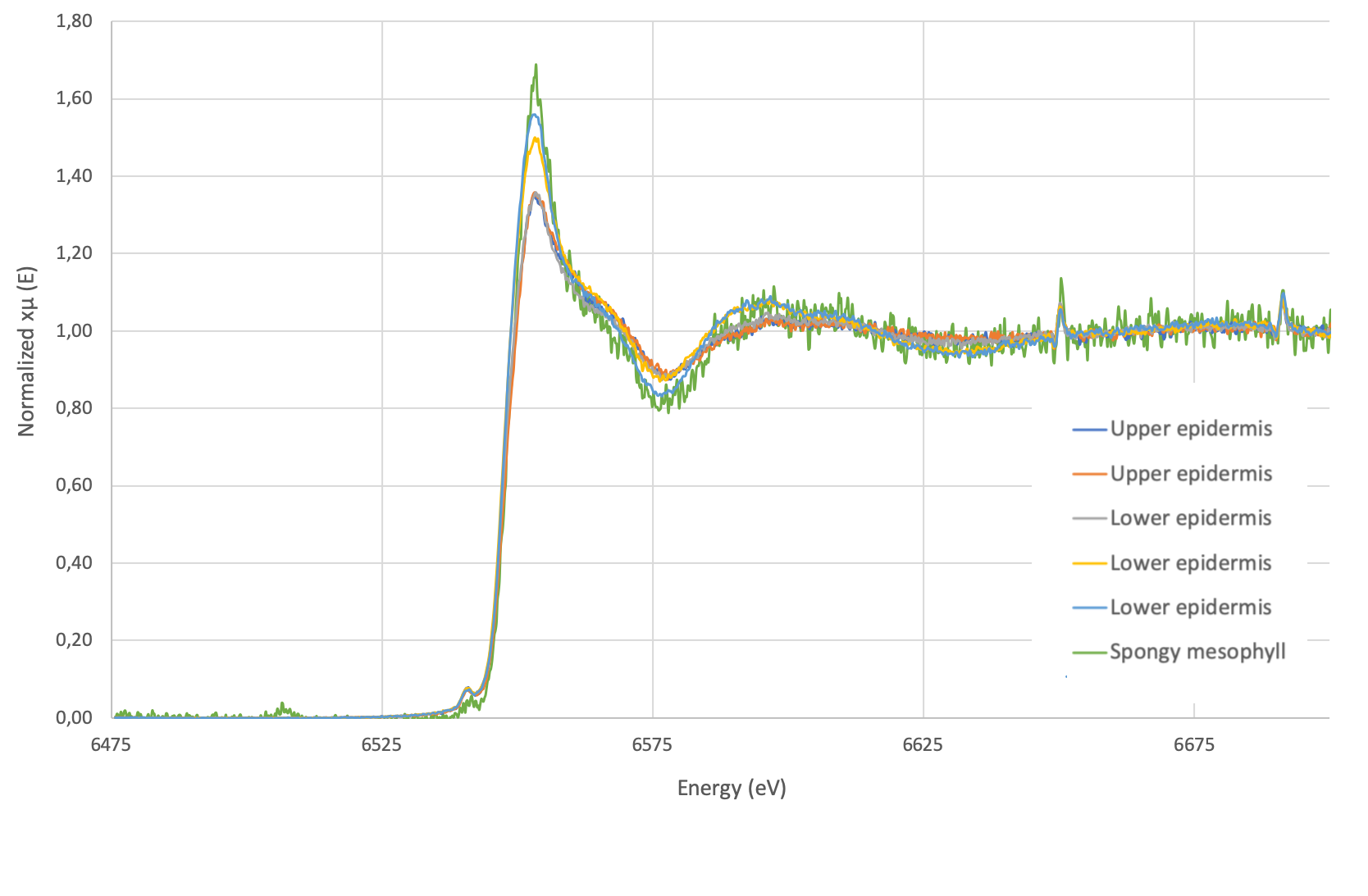


b)

a)

**Figure S13.** µXANES spectra at Mn K-edge obtained in different points of interest spotted among the upper epidermis, lower epidermis, spongy mesophyll, palisade mesophyll and vascular bundles of a) a frozen hydrated leaf mid-rid cross-section and b) a frozen hydrated leaf margin cross-section of transplanted G. meisneri
